# The Role of Emotion Projection, Sexual Desire, and Self-Rated Attractiveness in the Sexual Overperception Bias

**DOI:** 10.1007/s10508-021-02017-5

**Published:** 2021-08-13

**Authors:** Iliana Samara, Tom S. Roth, Mariska E. Kret

**Affiliations:** 1 Cognitive Psychology Unit, Department of Psychology, Leiden University, Wassenaarseweg 52, 2333 AK Leiden, the Netherlands; 2 Leiden Institute for Brain and Cognition, Leiden, the Netherlands; 3 Apenheul Primate Park, Apeldoorn, the Netherlands

**Keywords:** Sexual overperception bias, Error Management Theory, Speed-dating, Attraction, Social perception

## Abstract

A consistent finding in the literature is that men overperceive sexual interest in women (i.e., sexual overperception bias). Several potential mechanisms have been proposed for this bias, including projecting one’s own interest onto a given partner, sexual desire, and self-rated attractiveness. Here, we examined the influence of these factors in attraction detection accuracy during speed-dates. Sixty-seven participants (34 women) split in four groups went on a total of 10 speed-dates with all opposite-sex members of their group, resulting in 277 dates. The results showed that attraction detection accuracy was reliably predicted by projection of own interest in combination with participant sex. Specifically, men were more accurate than women in detecting attraction when they were not interested in their partner compared to when they were interested. These results are discussed in the wider context of arousal influencing detection of partner attraction.

**Supplementary Information:**

The online version contains supplementary material available at 10.1007/s10508-021-02017-5.

## Introduction

Almost half a century of research findings shows that men overperceive sexual interest in women (e.g., Abbey, 1982; Henningsen, 2004; Koeppel et al., 1993; La France et al., 2009; Levesque et al., 2006; Treat et al., 2015), a finding aptly termed as the “sexual overperception bias” (Haselton, 2003; Haselton & Buss, 2000). It has been suggested that this bias might rely on (1) projecting one’s own interest onto a given partner and (2) on the set of behaviors employed in partner selection (i.e., mating strategy) (Howell et al., 2012; Koenig et al., 2007). Recently, sex differences have been observed in these two factors, which revived the debate about the sexual overperception bias (Lee et al., 2020; Roth et al., 2021). Since this bias has been linked to the likelihood of sexual assault (Abbey et al., 1998), examining the factors relating to this bias has not only theoretical implications, but is crucial in illustrating the underlying causes for miscommunication in interpersonal relationships.

While on a date, with uncertainty running high, people can make two types of errors: They can see attraction when there is none or miss it when it is there. These errors are the focus of the error management theory (EMT; Haselton & Buss, 2000), an influential model explaining the sexual overperception bias. The EMT framework parallels statistical classification, in that inferring attraction when there is none (overperception) is a Type I error and missing attraction when attraction is indeed there (underperception) is a Type II error. Overperceiving attraction resembles a situation familiar to many chess players, in which a player is required to make a move even though any possible move would place her at a disadvantage (“Zugzwang”; Henningsen & Henningsen, 2010, p. 619). Similarly, a man believing that another is interested in him may feel bound to act; however, a move would place him at risk for social embarrassment. On the other hand, not noticing attraction when it is indeed present results in significant costs (i.e., a missed mating opportunity). Crucially, the costs associated with missing such a chance are asymmetrical across sexes (Haselton, 2003; Haselton & Buss, 2000). Men may suffer a greater cost if they miss a chance to reproduce (underperceive) than social embarrassment (overperceive). On the other hand, women expressing interest in a person not interested in a committed relationship may suffer costs due to missed paternal investment, according to the parental investment theory (Trivers, 1972). In conclusion, when detecting attraction, humans can either over- or underperceive attraction and each error is associated with specific costs, which shape the resulting baseline rates for detecting attraction in others.

People are generally not accurate in predicting attraction during dates (Veenstra & Hung, 2011). For example, a recent speed-dating study showed that participants were 51% accurate in correctly inferring whether their partner would be interested in another date with them (Prochazkova et al., 2019). Interestingly, participants responded in a manner similar to their own emotional state: Participants who were interested in their partner tended to indicate that their partner was also interested in them. This pattern, which we will refer to as the *projection mechanism*, has been suggested to drive the sexual overperception bias (Shotland & Craig, 1988) and has been supported by an emerging body of the literature (Henningsen & Henningsen, 2010; Koenig et al., 2007; Lee et al., 2020). Crucially, men tend to have greater levels of sexual interest in a given partner than women (Henningsen et al., 2006; Todd et al., 2007), which fits with the observed sex differences in sexual overperception. Nevertheless, despite the findings supporting the projection mechanism underlying the sexual overperception bias, it remains unclear whether men tend to project their own interest onto a given partner more than women (Lee et al., 2020; Roth et al., 2021).

Attraction does not emerge in a vacuum. Individual differences, such as sexual desire and self-rated attractiveness, likely shape how the overperception bias arises during an interaction (e.g., see Howell et al., 2012; Lee et al., 2020; Lemay & Wolf, 2016; Perilloux et al., 2012). The sexual overperception bias has been linked to men’s higher sex drive (Baumeister et al. 2001; see also Maner et al., 2005), suggesting arousal acts as a cue signaling that a mating opportunity should not be lost (Koenig et al., 2007). Indeed, emotional states have a significant impact on decision making (Damasio, 1996). Sexual arousal has been shown to increase the likelihood of risky sexual practices, likely indicating that inhibition is lowered during states of arousal (Ariely & Lowenstein, 2006; Skakoon-Sparling & Cramer, 2021; Skakoon-Sparling et al., 2015). Another likely factor in the sexual overperception bias is self-rated attractiveness. Specifically, people with higher self-rated attractiveness are more likely to report that a given partner is interested in them (Kohl & Robertson, 2014; Lemay & Wolf, 2016). This bias could be due to expectancies that self-rated attractiveness should match with others’ perception (Murray et al., 2000). Crucially, men rate themselves as more attractive than women (Hayes et al., 1999), which might explain the sexual overperception bias. Thus, these findings suggest that sexual desire and self-rated attractiveness are likely to influence the sexual overperception bias.

Speed-dating paradigms have been widely used to test sex differences in mate choice (e.g., Kurzban & Weeden, 2005; Lee et al., 2020). Speed-dating studies allow for the time- and cost-efficient investigation of the first moments of interaction (Finkel & Eastwick, 2008), as they create a space in which multiple people can have a brief date with multiple partners. Furthermore, speed-dates thus allow for the control of individual characteristics (e.g., mean attractiveness over many people, not a single data point). Importantly, speed-dating contexts create an ecologically valid setting to study sexual and romantic interactions, while maintaining a relatively controlled laboratory setting (Eastwick & Finkel, 2008; Finkel et al., 2007).

In an exploratory study, we employed a naturalistic speed-dating paradigm to investigate the effects of sex, own interest, sexual desire, and self-rated attractiveness on accuracy in detecting attraction. Based on previous evidence, we would expect that men exhibit lower attraction detection accuracy than women and that projection of own interest decreases attraction detection accuracy. Furthermore, we explored whether self-rated attractiveness and sexual desire scores influenced accuracy in detecting attraction.

## Method

### Participants

A total of 80 participants were recruited for a speed-dating event, 10 of which did not attend the experimental session. Furthermore, three participants (2 men) dropped out before the speed-dating started; resulting in a final sample of *N* = 67 (35 women; women: *M*_age_ = 22.03, *SD* = 2.26; men: *M*_age_ = 22.61, *SD* = 1.75). In total, 277 dates took place. All participants provided informed consent as according to the declaration of Helsinki. Participants were not compensated for their participation but received a complementary ticket to Apenheul Primate Park (Apeldoorn, the Netherlands). The procedure and methods were approved by the Leiden University Ethics Committee (CEP: 2020-02-20-M.E. Kret-V1-2169).

### Procedure

Participants first filled in questionnaires regarding demographic information; the 7-level Kinsey scale; Kinsey et al., 1948); self-rated attractiveness (7-point scale); and the Sexual Desire Inventory (SDI, Elaut et al., 2010; see Supplementary Material for Methods). Next, participants completed a battery of cognitive tasks (see Supplemental Material for full methods; preregistered using the AsPredicted database).^1^ Following completion of the tasks, participants went on 10 speed-dates (cf. Lee et al., 2020; Perilloux et al., 2012). Men and women sat at opposite sides of a table in a 2 × 2 fashion. Barriers were used to block the view of the opposite-sex participants. At the start of each date, participants were instructed to turn the barriers perpendicularly to separate each couple. Next, a bell rang, indicating the start of the date. After 4 min, the participants were asked to turn the barriers in a parallel fashion and indicate (1) how attractive they found their partner (7-point scale); (2) how attractive they considered them as a long-term mate (7-point scale); (3) whether they would be interested in going on another date with them (yes/no); (4) whether their partner would like to go on another date with them (yes/no); and (5) whether they knew each other (yes/no). The choice of asking participants to indicate whether they would like to go on another date with their partner (see also Asendorpf et al., 2011; Overbeek et al., 2013; Todd et al., 2007) instead of indicating sexual interest (as in Lee et al., 2020; Perilloux et al., 2012) was opted for given that it is more ecologically valid procedure. Participants were given 1 min to fill in the questionnaires. Male participants rotated from one partner to the next. After all opposite-sex couples had had a date, participants were thanked and debriefed.

### Statistical Analyses

To examine accuracy in detecting attraction, we calculated accuracy scores by comparing participants’ predictions regarding whether their partner would be interested in another date with them to the responses of their partners (0 = incorrect; 1 = correct). These accuracy scores were analyzed using Bayesian logistic multilevel modeling (MLM). The use of Bayesian MLM allowed us to account for the nested nature of the data, as well as examine the support for either the null or alternative hypothesis.

In total, we conducted 3 separate accuracy models. All models included accuracy scores as dependent variable and the fixed effect of sex. In the first model, we examined whether sex and own Interest influence accuracy scores by including the fixed effect of Own Interest, and its interaction with Sex. In the second model, we examined whether sex and sexual desire influence accuracy scores by including the fixed effect of sexual desire and its interaction with Sex. In the third model, we examined whether Sex and self-rated attractiveness influence accuracy scores by including the fixed effect of self-rated attractiveness and its interaction with Sex. All our binary predictors were sum coded (− 1 vs. 1), whereas all other predictors were scaled to obtain a mean of 0 and a standard deviation (*SD*) of 1.

An important benefit of Bayesian analyses is that they allowed us to place a prior on our assumptions, thus incorporating prior knowledge in the parameter estimation (Jeffreys, 1961; Lee & Wagenmakers, 2014). Given that uniform priors are considered improper in logistic models since they can bias the posterior distribution of the estimate (McInturff et al., 2004; Seaman et al., 2012), we opted for a Student’s *t* prior distribution with 7 degrees of freedom centered at 0 with *SD* of 1 (except for the intercept which had a *SD* of 10; Ghosh et al., 2018; see also Gelman et al., 2008). The use of Student’s *t* priors with 7 degrees of freedom has been recommended as opposed to other distributions, as it produces reliable estimates and reduces likelihood of computational estimation problems (i.e., “slow mixing Gibbs samplers”) even under conditions of separation (Ghosh et al., 2018, p. 362). Furthermore, an exponential prior with a *SD* of 1 was set for all error terms.

To facilitate the interpretation of the model coefficients, all estimates were exponentiated to obtain the odds ratio (OR). Effects were interpreted using the OR 95% highest density intervals (HDI), which summarize 95% of the posterior parameter distribution (Kruschke, 2018). If the 95% HDI spanned over 1, then the effect was not considered robust, given that this would suggest that accuracy spanned over 0.5 (i.e., chance level accuracy). To examine the reliability of interactions, we performed model comparisons to calculate Bayes factors (BF). As recommended, more than the default 1000 iterations per chain (1500) were set to allow for the efficient calculation of BFs. To test differences in interactions, we calculated a BF using the Savage–Dickey method (see Wagenmakers et al., 2010).

To further examine the direction of the errors associated with detecting attraction, we calculated a parameter *estimation* by subtracting the participants’ decision from their partners’ decision (see also Perilloux et al., 2012). This led to a parameter that took the values of 0 if the participants were accurate, 1 if they overestimated attraction, and -1 if they underestimated attraction. We then modeled this variable as a function of sex and own interest (i.e., whether the participant was interested in going on another date with his or her partner) (and their interaction) in an ordinal model. We opted for adjacent category models (ACM) with category-specific effects, which allowed us to detect differences between each category level (e.g., man vs. woman) for each of the potential outcomes. We set a prior of Student’s *t* with 7 degrees of freedom, scaled at 0 and with an *SD* of 2.

For all models, we followed the procedure outlined in the WAMBS checklist (Depaoli & van der Schoot, 2017). Trace and autocorrelation plots as well as posterior density histograms were examined. All analyses were conducted in R Studio (version 3.6.2) (R Core Team, 2019) using the brms package (Buckner et al., 2017, 2018; Bürkner & Vuorre, 2019).

## Results

A Bayesian chi-square test showed that men indicated more often than women that they were interested in going on another date with their dating partner (BF_10_ > 10; see Table 1), consistent with previous findings. Bayesian independent *t *tests showed that there was no difference between men and women in sexual desire (women: *M* = 50.71, *SD* = 12.19; men: *M* = 56.48, *SD* = 15.76; BF_10_ = 0.83) and self-rated attractiveness (women: *M* = 4.68, *SD* = 0.73; men: *M* = 5.03, *SD* = 0.68; BF_10_ = 1.46), contrary to previous findings (Lee et al., 2020; Perilloux et al., 2012).

**Table 1:** Percentage of men and women’s dating choice

	Women (%)	Men (%)
Yes	26	44
No	74	56

### Accuracy

**Fig. 1:**
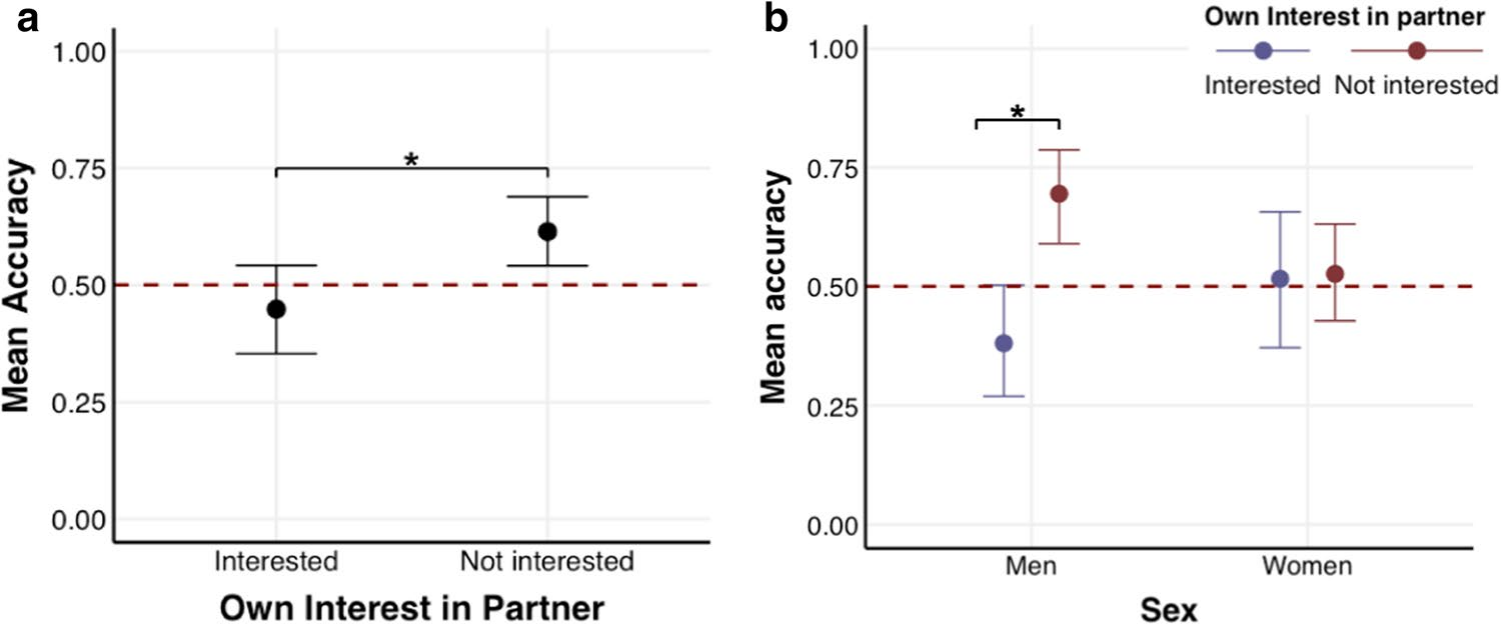
**a** Mean participant accuracy as a function of interest in their partner (interested vs. not interested). The figure shows that participants were less accurate when they were attracted to their partner than when they were not. **b** Interaction graph between sex and own interest. The figure illustrates that men were less accurate in detecting attraction in their partner when they were interested in the partner compared to when they were not interested. All error bars reflect 95% CrI, and the red line denotes chance accuracy level (.5)

In the first model, we examined whether sex and own interest influenced attraction detection accuracy (Table 2; Model 1). The results showed that overall participants were not able to reliably detect attraction. Own interest decreased accuracy (see Fig. 1a). Sex did not reliably predict attraction detection accuracy. We further examined whether the interaction between sex × own interest was reliable by comparing the more complex model (i.e., including the interaction) with a more parsimonious model (i.e., excluding the interaction). The calculated Bayes factor showed moderate evidence in favor of the complex model (BF_10_ = 7.39); indicating that the interaction was reliable. The interaction indicated that men were more accurate in detecting attraction when they were not interested compared to when they were interested in their partner (see Fig. 1b), whereas there was no difference in accuracy for women when they were interested in their partner compared to when they were not.

**Table 2:** Overview of all accuracy predicting models (1–3)

Predictors	Accuracy (Median odds ratios with 95% highest density intervals)					
	Model 1		Model 2		Model 3	
Intercept	1.14	[.86–1.52]	1.22	[.94–1.62]	1.16	[.88–1.53]
Sex	1.04	[.79–1.38]	1.03	[.78–1.36]	1.13	[.85–1.48]
Own interest	**.71**	**[.57–.88]**				
Sexual desire			1.23	[.96–1.58]		
Self-rated attractiveness					.82	[.64–1.05]
Sex × own interest	**.73**	**[.59–.90]**				
Sex × sexual desire			.84	[.66–1.07]		
Self-rated attractiveness × sex					1.09	[.86–1.39]
*Random effects*						
Var(Participant)	.37		.32		.33	
Var(Partner)	.26		.31		.31	

Reliable effects (OR 95% HDI not containing 1) are presented in bold

To further examine whether the reduced accuracy observed in men was driven by over- or underperceiving attraction when they were interested in their partner, we modeled the estimation variable as a function of sex × own interest. The model (Table 3; Model 1) showed that participants were more likely to accurately detect than underperceive attraction when they were interested in their partner compared to when they were not, consistent with the projection hypothesis. The coefficient for the interaction between sex × own interest in predicting spanned over 0, therefore, was not reliable.

**Table 3:** Overview of estimation predicting model as a function of sex and own interest

Predictors	Estimation (median estimates with 95% highest density intervals)	
	Model 1	
Intercept [under-accurate]	** − 1.63**	**[− 2.10 to − 1.18]**
Intercept [over-accurate]	**1.39**	**[.97–1.83]**
Sex [under-accurate]	.44	[.02–.87]
Sex [over-accurate]	.27	[**− **.14 to .65]
Own interest [under-accurate]	**.57**	**[.26–.90]**
Own interest [over-accurate]	**1.03**	**[.75–1.31]**
Sex × own interest [under-accurate]	** − **.09	[**− **.40 to .25]
Sex × own interest [over-accurate]	.28	[.01–.58]
*Random effects*		
Var (Participant)	1.06	
Var (Partner)	.59	

Reliable effects (95% HDIs not containing 0) are presented in bold

**Fig. 2:**
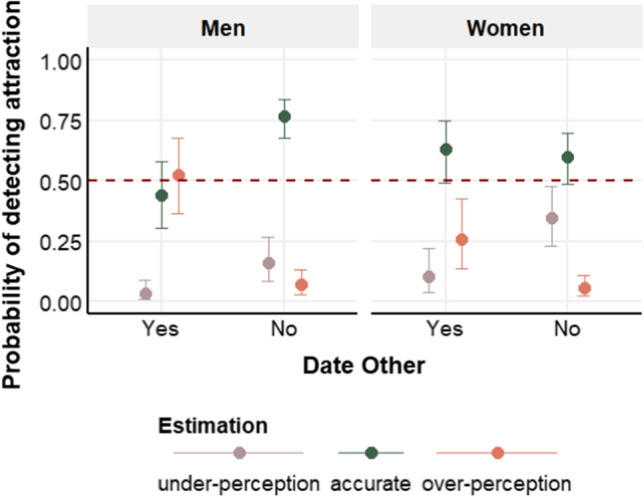
Interaction graph between sex and own interest showing the probability of each response category (i.e., underperception, accurate response, and overperception) for each factor level combination. The graph illustrates that men were more likely to overperceive attraction when they were interested in their partner compared to women and that men were more likely to accurately detect attraction than overperceive when they were not interested in their partner compared to women. Error bars reflect 95% CrI, and the red line denotes chance level (.5)

Regarding overperception, the coefficient of Sex was not reliable. Participants were more likely to overperceive than accurately detect attraction when they were interested in their partner than when they were not. The interaction between sex × own interest was not robust (Fig. 2). However, since our aim was to explore the difference between sexes in overperception of attraction, we conducted further point-null tests, which revealed that men were more likely than women to overperceive than accurately detect attraction when interested in their partner (BF_10_ > 10), whereas there was no difference between men and women when they were not interested in their partner (BF_10_ = 0.95).

In the second accuracy model, we examined the effect of trait sexual desire (i.e., sexual desire scores) and its interaction with Sex. All coefficients spanned over 1, therefore, were not robust. In the third model, we examined the effect of self-rated attractiveness and its interaction with Sex. All coefficients spanned over 1, therefore, were not robust.

### Baseline Differences Accounting for Accuracy Differences

In the previous analyses, we observed that men were more likely to accurately detect attraction in their partner if they were not interested in their partner, which could be due to differences in state arousal levels influencing their decision making. An alternative explanation, however, could be that men guessed their partner’s response, and given that women overall tend to respond more often in the negative, it coincidentally ended up matching, leading to increased accuracy.

To examine this, we conducted a Bayesian binomial test using the BayesianFirstAid package (Bååth, 2013). The number of successes in detecting attraction was calculated only for instances where male participants were not interested in their partner and indicated that their partners were not interested in them. If men were indeed guessing when they were not interested in their partners, then the probability of success (i.e., a correct response) should be approximately close to 0.5 accuracy (i.e., chancel level). The results of the Bayesian binomial test showed that men were more likely to correctly indicate that their partners were interested in them (relative success frequency: 0.74, 95% HDI [0.66, 0.81]). It should be noted that if men had prior knowledge of the average positive response rates for women (e.g., because of prior dating experience), they would be able to accurately guess their responses 61% of the time (0.26 × 0.26 + (1–0.26) × (1–0.26) = 0.61; see also Place et al., 2009 for a similar approach). Since 0.61 was outside of the 95% HDI range, it can be inferred that men indeed were more likely to accurately detect attraction in their partner rather than guessing.

## Discussion

The present study explored the effects of sex, own interest, sexual desire, and self-rated attractiveness in the overperception bias using a naturalistic speed-dating paradigm. Overall, we found that men were more willing to go out with their partner as compared to women. Importantly, our findings illustrate that projection of own interest influences attraction detection, particularly in men. Specifically, men were more accurate in detecting attraction if they were not interested in their partner compared to when they were. Furthermore, when men were interested in their partner, they overperceived interest more than women. However, there was no difference between sexes when participants were not interested in their partner. Women were approximately 50% accurate in detecting attraction, independent of whether they were interested in their partner or not. Sexual desire and self-rated attractiveness did not influence accuracy in detecting attraction. In the section below, we discuss these results in more detail.

First, we found that men were more likely to indicate that they were interested in going out with their partner again compared to women. This is in line with previous literature across different countries and target samples (i.e., university students and general population) showing a consistent pattern in terms of reduced male selectivity (e.g., Asendorpf et al., 2011; Fisman et al., 2006; Kurzban & Weeden, 2005; Lenton & Francesconi, 2010; McClure et al., 2010; Overbeek et al., 2013; Todd et al., 2007). An explanation could be that men wanted to maximize the number of dates that they could get, consistent with EMT (Haselton & Buss, 2000) which suggests that missing a dating opportunity could be more costly for men than for women. Also, the low likelihood of women indicating that they would like to meet their partner again supports previous findings showing that women are typically choosier than men (Todd et al., 2007; Trivers, 1972). In conclusion, we show that men were more likely than women to decide that they would like to go on another date with their partner supporting the notion that men are slightly less picky regarding dating.

It might be argued that the increased tendency of men to respond positively after a date can be explained by the fact that only men had to rotate between partners in our study. This effect was described by Finkel and Eastwick (2009), who showed that the reduced selectivity is nullified when female participants also rotate between partners. However, a recent meta-analysis showed that the female choosiness effect is robust across studies, and that the rotation effect did not moderate female choosiness (Fletcher et al., 2014), nor has been replicated (e.g., Overbeek et al., 2013). It is therefore unlikely that the partner-rotation effect can explain our findings. Nonetheless, future research should examine whether the sex-rotation-setup modulates the relationship between sex and the sexual overperception bias.

Interestingly, we found that men were more accurate when they were not interested in their partner compared to when they were, whereas women were approximately at 50% independent of their interest in their partner. An explanation for this interaction between sex and projection of own interest might be because of a link between choice biases and physiological arousal. Previous research has shown that men can detect changes in genital arousal that indicate sexual arousal within five minutes, and importantly, the correlation between genital arousal and subjective sexual arousal is reliable for men, but not for women (Kukkonen et al., 2007; see also Dekker & Everaerd, 1988). Physiological arousal influences our affective state, which can in turn bias our decisions (Damasio, 1996; see also Storbeck & Clore, 2008). For example, men that were shown sexually arousing stimuli were more likely to indicate that attractive women were sexually aroused than not (Maner et al., 2005) and sexually aroused participants are more likely to engage in risky sexual practices (Ariely & Lowenstein, 2006; Skakoon-Sparling & Cramer, 2021; Skakoon-Sparling et al., 2015). Thus, our findings might suggest that in situations where men were not interested in their partner, this biasing emotional state was not present, thus allowing them to accurately detect that their partner is not interested in them. Indeed, previous research has suggested that cues signaling disinterest might be easier to detect than cues signaling interest, especially in zero-order acquaintance settings (Hall et al., 2015). Given that the concordance between bodily and subjective arousal is not as robust in women, it is not surprising that women were not necessarily biased as much as men in terms of detecting attraction. In conclusion, our findings extend previous evidence showing that accuracy does not only depend on sex or projecting one’s own emotion on a partner, but accuracy is in fact dependent on an interplay between these two factors.

The estimation model complemented the results of the accuracy models. Interestingly, we found that both men and women were likely to overperceive attraction when they were interested in their partner compared to when they were not. Crucially, when men were interested in a partner, they overperceived interest more than women, which likely explains the decreased accuracy exhibited in men. These findings are partially consistent with EMT (Haselton & Buss, 2000). EMT predicts that men would be more likely to overperceive attraction than women. However, our findings highlight that perhaps the effect of being attracted to a given partner should be incorporated as an additional parameter in EMT (Lee et al., 2020), because if men are not interested, they are in fact very likely to be accurate regarding attraction. Thus, our findings support and further extend the EMT framework by showing that the addition of interest in a given partner might be crucial in predicting overperception.

Curiously, we found no effect of sexual desire on attraction detection accuracy. Our results are inconsistent with previous findings (Lee et al., 2020; Perilloux et al., 2012). One reason for this discrepancy could be that previous studies focused on short-term mating strategies, whereas we examined overall sexual desire. It is well known that sociosexuality—the inclination to form short-term relationships (Kinsey et al., 1948)—differs between men and women (Clark & Hatfield, 1989). Importantly, given that sexual desire and sociosexuality are highly correlated (O’Connor et al., 2014), we expected to observe similar findings as Lee et al. (2020). However, in our dataset we found no difference in sexual desire between sexes, whereas in Lee et al. (2020) sociosexuality was significantly higher for men than women (see also Roth et al., 2021). Either due to the differences in instruments or the differences in sample characteristics, we did not find an effect of sexual desire on attraction detection accuracy. Future research should investigate the effect of sexual desire and its association with sociosexuality and sex on attraction detection accuracy.

In addition, we found no effect of self-rated attractiveness on accuracy, in contrast with previous research (Lee et al., 2020; Perilloux et al., 2012). A potential explanation for this finding could be that in the present study, we examined physical attractiveness exclusively. We could therefore only speculate that our sample was similar to previous research in terms of other factors that can constitute attractiveness (e.g., personality). Nevertheless, previous research has shown that personality has negligible effects on both men and women’s desirability (Kurzban & Weeden, 2005). Furthermore, self-rated attractiveness has been found to play a role in overperception together with short-term mating styles (Howell et al., 2012; see also Lee et al., 2020; Perilloux et al., 2012). However, in our sample, most participants indicated they were searching for a long-term relationship. Thus, this pronounced long-term relationship focus might have prevented the interplay between self-attractiveness and mating strategy to emerge.

One crucial point that cannot be disentangled in the context of the present study is whether women and men interpreted the question regarding the wish to go on another date with their partner similarly. Specifically, in previous studies, participants were asked to indicate how sexually interested they were in their partner (Lee et al., 2020; Perilloux et al., 2012). However, in the present study, participants were asked to indicate whether they would like to go on another date with their partner (see also Asendorpf et al., 2011; Overbeek et al., 2013; Todd et al., 2007 for similar setups). It could be argued that this question led female participants to respond to the perceived question of “Are you romantically interested in your partner?” and male participants to respond to the question of “Are you sexually interested in your partner?” Even though this cannot be tested in the present study, it is quite likely that the response pattern would have remained the same. Previous research has shown that romantic interest and sexual interest follow the same sex differences, where women are choosier than men (Fletcher et al., 2014). Crucially, asking about the wish to go on another date rather than sexual interest is a strength of the current study, as it increases its ecological validity, given that it resembles real-life situations more closely (e.g., online dating sites; see Kurzban & Weeden, 2005).

It should be noted that in the present study, we examined only heterosexual participants; therefore, our findings cannot be directly generalizable to non-heterosexual populations. Furthermore, our sample consisted predominantly of university students. University students offer a prime target sample for sexuality research given the greater interaction frequency with opposite-sex partners and the increased necessity to infer sexual interest (Perilloux et al., 2012) and are commonly the primary target for such studies (e.g., Lee et al., 2020). Importantly, most participants in our study were interested in a committed relationship (only 2 participants were not), which limited our ability to investigate whether different mating strategies might influence attraction detection accuracy (e.g., Lee et al., 2020; Perilloux et al., 2012). Crucially, a limitation that stems from the use of a speed-dating setup is that we cannot assess whether the personality characteristics and social skills of our sample are representative of a wider population (Finkel & Eastwick, 2008). Future research should investigate more heterogeneous samples in terms of educational background and age.

The current study shed light on several factors that underlie the sexual overperception bias. Given that this bias is linked to the likelihood of assault (Abbey et al., 1998), the study’s findings are crucial in elucidating and reducing miscommunication between the sexes in dating contexts (Perilloux et al., 2012). Crucially, we showed that sex and projection of own interest are intertwined and should not be seen as competing, but rather as complementary explanations. Importantly, our findings cast doubt on previous research suggesting that one’s own interest, sexual desire, and self-rated attractiveness might fully explain the sexual overperception bias (Lee et al., 2020; see also Roth et al., 2021). Therefore, our results not only support the EMT framework, but further suggest that the incorporation of sex differences in projection of own interest might be a useful addition to the EMT framework.

## Supplementary Information

Below is the link to the electronic supplementary material.

Supplementary file1 (DOCX 20 KB)

## Data Availability

All materials (data and code) are uploaded on Dataverse (10.34894/6UVDBN).

## Footnotes

1 Reference number #36,394.
