# Supplementary material for: The Role of Emotion Projection, Sexual Desire, and Self-Rated Attractiveness in the Sexual Overperception Bias
Source: Arch Sex Behav. 2021 Aug 13;50(6):2507–16. doi: 10.1007/s10508-021-02017-5 (PMC8416843; doi:10.1007/s10508-021-02017-5)
Supplement: Supplementary file 1 — Supplementary file1 (DOCX 20 KB) [file 10508_2021_2017_MOESM1_ESM.docx]

**Supplemental Material**

**Methods**

Participants registered for the experiment using an online form (Qualtrics). They were asked to provide informed consent and indicate that they met the inclusion criteria. After providing informed consent, participants were divided in 4 groups of 20 (10 women) and indicated their preferred timeslot. Upon arrival to the lab, participants were asked to sign an informed consent. Next, participants received a unique ID, submitted the olfactory stimuli and filled in 3 questionnaires (a) demographic information; b) 7-level Kinsey scale; Kinsey, 1948); c) Sexual Desire Inventory, SDI, Elaut et al., 2010). Next, a researcher took portrait pictures of

the participants (ID photos, Puts et al., 2012) whereas, another researcher collected the audio stimuli (Dutch equivalent of RAINBOW passage; van Lierde et al., 2001) using a Shure V5

microphone.

Following stimulus collection, participants performed a battery of cognitive tasks. Specifically, participants were asked to perform a dot-probe (van Rooijen et al. 2017), effort (Hahn et al. 2013), and preferential looking task (Leder et al. 2016) to measure visual attentional biases, and three rating tasks (i.e., visual, auditory, and olfactory). The task section of the study lasted approximately one hour. After all participants had completed the tasks, they were led into the speed-dating room to conduct 10 speed-dating sessions. Each speed date lasted for 5 minutes. Both individuals were videotaped during the date. After each date, they indicated a) how attractive they found their partner (7-point scale); b) how suitable they found their partner as a long-term romantic partner (7-point scale); c) how attractive they believed their partner perceived them to be (7-point scale); d) how suitable their partner perceived them to be as a long-term romantic partner (7-point scale); e) whether they would like to go on another date with their partner (yes/no); and f) whether they believed their partner would like to go on another date with them (yes/no). The speed-dating section of the study lasted approximately one hour. After the study was completed, participants were asked to give consent for use of their stimuli and contact information, debriefed, and given a complementary ticket to Apenheul Primate Park (Apeldoorn, the Netherlands).

**Tasks**

*Dot-probe task*

The dot-probe task is a well-known measure of cognitive biases (see van Rooijen et al., 2017). In this task, two images are briefly presented on the left and right center locations of the display and then, replaced by a dot on one of the two locations. The pictures collected by participants were used as stimuli. In each trial, participants viewed a combination of the pictures of opposite-sex individuals (45 combinations). Each trial started with a centrally presented fixation cross that remained on the display until response. Then, the two images appeared briefly on either the left or right location on the display, and were subsequently replaced by a dot. Participants were instructed to indicate the location of the dot using the corresponding keyboard key (*z* for left; *m* for right). The task started with 10 practice trials, after which participants saw all combinations of the stimuli twice (90 trials), leading to a total of 100 trials. The task lasted approximately 6 minutes.

*Effort task*

The effort task was modeled after Hahn and colleagues (2013). Participants saw a picture of the 10-opposite sex individuals per trial. Each trial had a standard presentation time of 4 sec; however, participants could increase or reduce the duration (100-ms steps) by pressing the corresponding key combination (z-x for reducing, n-m for increasing duration). The task started with 5 practice trials, after which participants viewed all pictures of the opposite-sex individuals 4 times, leading to a total of 40 trials. Participants were instructed that their responses would not influence the total task duration to minimize the likelihood that they would purposefully try to reduce stimulus duration. In total, the task lasted approximately 8 minutes.

*Preferential looking task*

The preferential looking task was modeled after Leder and colleagues (2016). Participants saw combinations of all the pictures of the opposite-sex participants (45 trials). Each combination was presented for 3 sec. The task lasted approximately 6 minutes.

*Visual rating task.*

Participants viewed all pictures of the opposite-sex individuals. They were asked to indicate a) how attractive they found they depicted individual and b) how suitable they found them as a long-term romantic partner. The task lasted approximately 5 minutes.

*Auditory rating task.*

Participants listened to all audio-clips of the 10 opposite-sex individuals. They were asked to indicate a) how attractive they found they voice of the individual and b) how suitable they found them as a long-term romantic partner. The task lasted approximately 7 minutes.

*Olfactory rating task.*

Participants were asked to smell the olfactory stimuli (i.e., t-shirts) of the 10 opposite-sex individuals. The stimuli were presented through opaque containers, so low-level features (e.g., color of shirt) would not influence their rating. They were asked to indicate a) how attractive they found they voice of the individual and b) how suitable they found them as a long-term romantic partner. The task lasted approximately 7 minutes.
